# Pathomorphological Features of the Novel Coronavirus Disease in Patients with Systemic Amyloidosis

**DOI:** 10.3390/biomedicines11102811

**Published:** 2023-10-17

**Authors:** Liudmila Mikhaleva, Zarina Gioeva, Valery Varyasin, Elvira Berezhnaja, Rositsa Vandysheva, Nikita Gutyrchik, Valentina Pechnikova, Andrej Kontorshchikov, Konstantin Midiber, Lev Kakturskij

**Affiliations:** 1 Avtsyn Research Institute of Human Morphology, Federal State Budgetary Scientific Institution “Petrovsky National Research Centre of Surgery”, 117418 Moscow, Russia; mikhalevalm@yandex.ru (L.M.); rositsamok@gmail.com (R.V.); gyt94@yandex.ru (N.G.); valiagtx@yandex.ru (V.P.); andreistr.ru@mail.ru (A.K.); midiberkonst@gmail.com (K.M.); levkaktur@mail.ru (L.K.); 2 City Clinical Hospital No. 52, 123182 Moscow, Russia; pao52@mail.ru (V.V.); dr.berezhnaia@gmail.com (E.B.); 3 Medical Institute, Peoples’ Friendship University of Russia (RUDN University), 117198 Moscow, Russia

**Keywords:** amyloidosis, COVID-19, SARS-CoV-2, comorbid pathology, immunohistochemistry

## Abstract

Amyloidosis is one of the rare systemic illnesses characterized by the deposition of amyloid fibrils in various organs and tissues. There is a common point between COVID-19 and systemic amyloidosis regarding the multiorgan involvement in the pathological process which leads to a heightened risk for severe morbidity and mortality in amyloidosis patients who contracted COVID-19. We performed a pathomorphological analysis of the autopsy records of 22 patients who had COVID-19 and pre-existing systemic amyloidosis. The premortem diagnosis of systemic amyloidosis was established in 55% of patients, and in other 45% of cases, amyloidosis was found at autopsy. Based on the results of immunohistochemical amyloid typing, amyloid A (AA) amyloidosis was detected in 23%, amyloid light chain (AL) lambda in 32%, AL kappa–in 9%, and transthyretin (ATTR) amyloidosis–in 36% of observations. Immunohistochemical staining with an antibody against SARS-CoV-2 Spike (S) protein revealed positive immune reactions in type II alveolocytes in 59% of deceased persons. The analysis of autopsy findings indicates that patients with systemic amyloidosis are more likely to experience an aggressive clinical course of COVID-19 which leads to a multiorgan failure and a higher risk of fatal outcome.

## 1. Introduction

The novel coronavirus disease (COVID-19) pandemic became an unprecedented global challenge that affected all aspects of human life and caused colossal social, economic, and human losses. SARS-CoV-2 is a single-stranded ribonucleic acid (RNA) enveloped virus belonging to the family of Coronaviridae (Betacoronavirus, lineages B and C, including SARS-CoV and MERS-CoV which induced outbreaks with high fatality rates), ranging from 60 to 140 nm in diameter and possessing 9–12 nm in length crown-like spikes [1,2,3].

During the pandemic, the predominance of most aggressive SARS-CoV-2 variants was associated with such severe clinical manifestations of the disease as bilateral interstitial pneumonia, acute respiratory distress syndrome with diffuse alveolar damage (DAD) and various types of extrapulmonary disorders, including thrombotic/thromboembolic events, myocarditis, vasculitis, etc. [4,5,6,7].

Extrapulmonary organ injury from COVID-19 can often become the major cause of death. The disease-posed threat is linked to pre-existing comorbidities that are common in elderly patients and are considered a predictor of poor outcomes [8,9,10].

COVID-19 has been shown to cause even more serious implications in patients with systemic amyloidosis–a rare multiorgan disease involving the heart, kidneys, liver, gastrointestinal tract, and nervous system [11,12,13,14]. Patients with AL amyloidosis represent a particularly vulnerable population, especially those on active treatment with chemotherapy, as they have a lower immune response and are likely to be at a higher risk for contracting COVID-19. Ultimately, they have a more rapidly progressive and severe clinical pattern of the disease exhibiting multisystem involvement and multiorgan complications. In addition, patients with amyloidosis may be more susceptible to toxicities of drugs used to manage COVID-19 which makes a significant contribution to a patient’s severe clinical course of the disease [15,16].

In some of the autopsy studies performed during the COVID-19 pandemic, amyloidosis was found in patients who remained undiagnosed during their life. This happened because the clinical symptoms of amyloidosis are not specific and often mimic those of other conditions. Clinical management of COVID-19 in such patients does not take into consideration the clinical features of amyloidosis which can often lead to severe complications and deaths.

The objective of this study is to present the pathomorphological findings collected from deceased persons with severe course of COVID-19 overlapping with systemic amyloidosis.

## 2. Materials and Methods

Autopsies were performed at the Pathology Department of the State Budgetary Healthcare Institution of Moscow–City Clinical Hospital No. 52 of Moscow Healthcare Department with close adherence to the biosafety rules and in compliance with the WHO guidelines and the regulatory requirements of the Ministry of Health and the Federal Service for Surveillance on Consumer Rights Protection and Human Well-being of the Russian Federation [17]. The method of real-time polymerase chain reaction (RT-PCR) was used to detect SARS-CoV-2 RNA on pre- and post-mortem samples collected from all deceased subjects. 

Postmortem examinations were carried out no later than 1.5 days after the pronouncement of death. A complete autopsy was performed in all cases. Fixation in 10% neutral buffered formalin solution and paraffin embedding was used to preserve the obtained tissue samples. Then, a histological examination of the lungs, heart, liver, kidneys, and spleen was carried out. All histological sections were stained with Mayer’s hematoxylin plus eosin (H&E) reagent (BioVitrum company, St. Petersburg, Russia). Congo red stain (CR) was used for detecting amyloid deposits (BioVitrum company, St. Petersburg, Russia). To examine the samples, we used the polarized light microscope equipped with a set of fluorescent filters 340–560 nm (Leica DM2500, Leica Microsystems AG, Wetzlar, Germany).

For conducting IHC analysis, tissue sections were prepared of 3–5 µm thickness from paraffin blocks. The antibody panel included a SARS-CoV-2 (COVID-19) Spike RBD antibody [HL257] GTX635692 (1:500; GeneTex, Irvine, CA, USA), and for amyloid typing: polyclonal antibodies to amyloid P-component (1:1000; Cloud-Clone Corp., Katy, TX, USA) and transthyretin (1:500; Cloud-Clone Corp., Katy, TX, USA), monoclonal antibodies to AL kappa (1:200; Clone CH15, Leica Biosystems, Novocastra, United Kingdom), AL lambda light chains (1:200; Clone SHL53, Leica Biosystems, Novocastra, United Kingdom), and AA amyloid (1:500; Clone C3, Cloud-Clone Corp., Katy, TX, USA) on the Leica BOND-MAX IHC staining system.

## 3. Results

We evaluated the results of autopsies performed on 22 decedents with systemic amyloidosis who died from severe COVID-19 in Moscow from 20 April 2020 to 15 November 2022. The study included 11 males and 11 females aged 53 to 95 years (mean age of all patients—77; men—77.2, women—78.2 years). The length of stay at the hospital ranged from 2 to 32 days, and the interval between the disease onset and hospitalization was not more than 5 days from the appearance of the first symptoms. Pre-mortem PCR tests for detecting SARS-CoV-2 RNA were performed in the certified laboratories, and the results showed that all 22 tested patients had COVID-19 infections. In autopsy material, the PCR-confirmed presence of SARS-CoV-2 RNA was reported in 15 (68%) of 22 cases.

In 14 patients, COVID-19 caused by the SARS-CoV-2 virus was identified as a primary disease, and systemic amyloidosis as a pre-existing comorbidity. In 8 other patients, the coronavirus infection was deemed to be a comorbidity occurring alongside the following underlying disorders: systemic amyloidosis (n = 3), atherosclerotic encephalopathy with age-related amyloidosis considered a concomitant disease (n = 2), multiple myeloma with the secretion of monoclonal light chains amid the development of systemic amyloidosis as a pre-existing disease (n = 1), hypertensive disease with prevailing renal and cardiac manifestations complicated by the development of systemic amyloidosis (n = 1), and chronic interstitial nephritis complicated by the development of systemic amyloidosis (n = 1). Of 22 described cases, the premortem diagnosis of systemic amyloidosis was established in 12 (55%) patients, and in 10 (45%) cases amyloidosis was found at autopsy. 

### 3.1. Lung and Upper Airway Findings

The macroscopic assessment of pathology specimens demonstrated that all deceased persons had no obstruction or abnormalities of the laryngeal lumen. The trachea had pink dull mucosa, which in 3 cases was stippled with punctate hemorrhages. Commonly, the bronchial lumen was patent, and the mucosa appeared pale pink or reddish grey in color. In two cases, the tracheal and bronchial lumens contained thick yellow mucus, and in one case the residual amount of “coffee grounds” was found. 

In most deceased persons the lungs were larger and heavier than normal, weighing from 1100 to 2050 g. Changes in the lungs included an increased firmness and significantly reduced aeration. The external aspect of the lungs was of purple-red or dark-cherry coloration. In some of the autopsy cases, foci of pulmonary consolidation were found in both lungs. On the cut surface, the pulmonary parenchyma had a dark red appearance with marked congestion and sometimes brown/grey mottled color. In 12 (55%) cases, the walls of bronchi were dense, overhanging the cut surface. The autopsy also revealed focal pulmonary hemorrhages in 5 (23%) fatalities. In 19 (86%) examined cases, the visceral pleura was not involved in the pathological process, remaining smooth and glossy. In one case (4.5%), a pronounced pleural anthracosis of both lungs was detected, and in two cases (9%) pleural adhesions on the interlobar surfaces of the lungs were found. 

**Figure 1:**
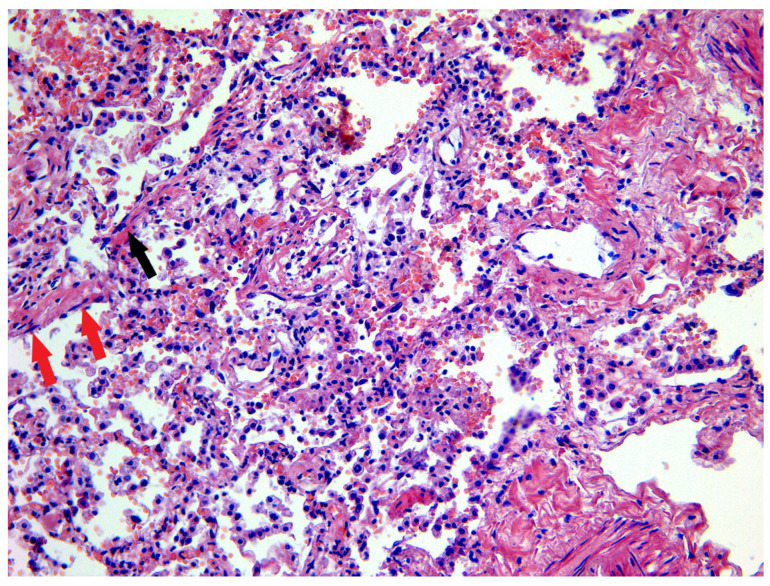
Microscopic sample of lung tissue of the deceased male patient, 59 years of age, with PCR-confirmed COVID-19. Day 17 from the illness onset. Hyaline membranes along the alveolar contours (black arrow), individual fibroblasts in the alveolar lumens (red arrow), congestion and edema of the interalveolar septa. Staining with H&E, ×200.

Microscopic examination of the pulmonary tissue revealed edema and congestion of the interalveolar septa, and hyaline membranes were identified along the alveolar contours (Figure 1). 

The alveolar lumens contained neutrophils, macrophages, siderophages, lymphocytes, plasmocytes, erythrocytes, fibroblasts, giant cells, and large type 2 alveolocytes of irregular shape. Desquamation of the alveolar epithelial cells was present in most observations (86%), and in some of them, the normal alveolar epithelium was replaced by squamous metaplastic epithelial cells. Pulmonary infarctions in different locations were observed in two patients (9%). Fibrinous pleuritis was found in two cases and pleural hyalinosis–in one case. Microvascular thrombosis was found in 16 deceased persons (73%), the thrombi were mostly localized in medium-small caliber vessels of the lungs (Table 1). 

Post-mortem assessment of the specimens demonstrated features of the exudative phase of DAD in 9 (41%) cases, and the proliferative phase of DAD in 13 (59%) cases.

**Figure 2:**
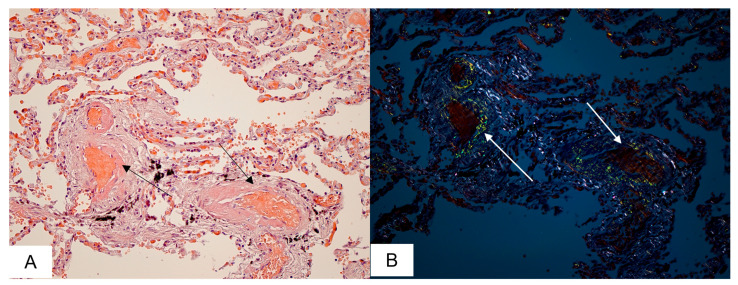
Microscopic sample of lung tissue of the deceased male patient, 86 years of age. Day 11 from the illness onset. Microscopic examination demonstrates amyloid deposits within the vascular walls localized in the pulmonary parenchyma(black arrows) (**A**) displaying apple-green birefringence under polarized light(white arrows) (**B**). Staining with CR, ×200.

Amyloid deposits in the lungs were found in 8 (36%) autopsy cases. Homogenous eosinophilic masses were detected in the interalveolar septa, within the alveolae and vascular walls. After staining with CR, the amyloid deposits demonstrated characteristic apple-green birefringence in polarized light (Figure 2).

### 3.2. Cardiovascular System Findings

Gross examination showed that the heart weight ranged from 300 to 700 g. The cardiac cavities were dilated in 11 (50%) cases. Hypertrophy of the left and right ventricles was found in 18 (81%) and 8 (36%) deceased persons, respectively. The most marked myocardial hypertrophy was observed in a patient with the underlying diagnosis of multiple myeloma. The walls of the left and right ventricle were 3.0 and 0.9 cm thick, respectively. The heart weight was 640 g. Other findings included a narrowing of the heart chambers, thickening of the endocardium, and the dense yellowish-brown myocardial muscle with multiple small-sized scars. Pericardial effusion occurred in 5 (23%) cases, and in one of them, it was associated with massive hemorrhages in the epicardium. Post-mortem examination of the aorta in most cases revealed an increased density of its walls and the presence of yellow spots and stripes. 

Microscopically, the myocardium was characterized by dystrophic changes, fragmentation, and focal necrosis of cardiac myocytes, as well as sclerosis and hypertrophy of the vascular walls. Focal cardiosclerosis was determined in 15 (68%) cases, hypertrophic and atrophic cardiac myocytes in 17 (77%) and 7 (32%) cases, respectively. The features suggestive of myocarditis were found in four (18%) deceased persons. 

In 19 (86%) cases, autopsy findings included amyloid deposits in the heart, and homogenous eosinophilic masses localized both in the vascular walls and myocardial stroma. The most remarkable diffuse and focal interstitial/intravascular amyloid deposits, surrounded by clusters of cardiac myocytes with dystrophic or atrophic changes were identified in patients with the underlying diagnosis of multiple myeloma, periodic disease, or systemic amyloidosis.

### 3.3. Liver Findings

Grossly, the liver was smooth and firm with a weight ranging from 1000 to 3500 g. The cut surface of the liver had a brown or yellowish-brown color with a nutmeg appearance. Light microscopy showed central vein and sinusoidal congestion, disarrangement of hepatic cell plates at the center of hepatic lobules, atrophy, and in some cases–necrosis of the centrilobular hepatocytes and focal fatty degeneration with lipid droplets. 

**Figure 3:**
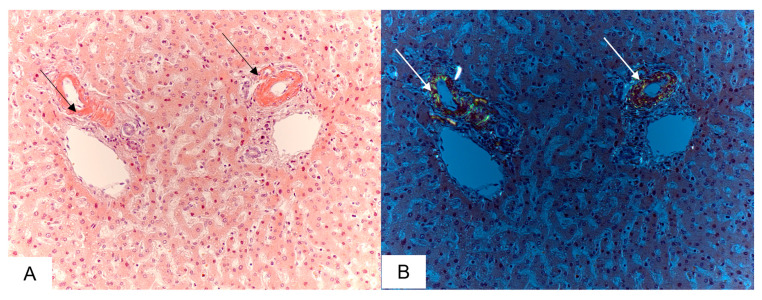
Microscopic sample of the liver tissue of the deceased female patient, 80 years of age. Day 8 from the illness onset. Amyloid deposits in the hepatic portal tract (black arrows) (**A**); amyloid masses displaying apple-green birefringence under polarized light (white arrows) (**B**). Staining with CR, ×200.

Amyloid deposition in the liver, involving the portal triads and vascular walls was detected in 10 (45%) cases (Figure 3). 

Round cell infiltration was observed closely to the portal tracts. Autopsy findings in a patient with multiple myeloma included irregular sclerosis of the portal stroma with focal lymphocytic infiltration and multiple fields of infiltration by atypical plasma cells which extended outside the terminal plate and contained focally deposited amyloid in the spaces between hepatic plates and within the vascular walls. 

### 3.4. Kidney Findings

On gross examination, the kidneys in most cases appeared firm, grey or pale-brown colored with a fine granular surface, weighing from 100 to 440 g. Their capsule was easily removed. In 12 (55%) autopsies, the border between the renal cortex and outer medulla was not clearly distinguishable. In all observations, the pelvicalyceal system was not dilated. Thin-walled cysts filled with clear yellow fluid were found in 5 (28%) cases.

Microscopy demonstrated dystrophic changes in the renal tubular epithelium which sometimes was coupled with necrosis; arterial sclerosis and hyalinosis; and marked glomerular capillary congestion. Other findings included partial glomerular sclerosis and hyalinosis along with intact hypertrophied glomeruli, and tubular autolysis. 

**Figure 4:**
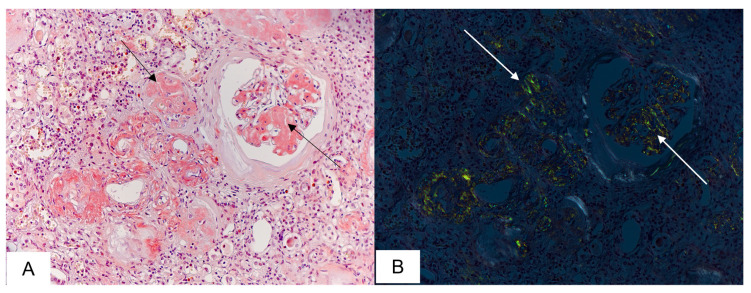
Microscopic sample of renal tissue of the deceased male patient, 78 years of age. Day 9 from the illness onset. Microscopic features of renal amyloidosis. Amyloid depositions in the glomeruli, vascular walls, and stroma (black arrows) (**A**), characteristic apple-green birefringence under polarized light (white arrows) (**B**). Staining with CR, ×200.

Amyloid deposits in the kidneys were identified in 16 (73%) deceased persons. In 6 of them, the total glomerular network was affected by amyloid deposition with marked interstitial fibrosis and round cell infiltration. In some cases, the renal tubules and collecting ducts contained lumpy and irregular eosinophilic amyloid masses; the arteriolar walls were thickened by amyloid deposits. Hyaline casts in the tubular lumens were found almost in all autopsies (Figure 4). 

### 3.5. Spleen Findings

**Figure 5:**
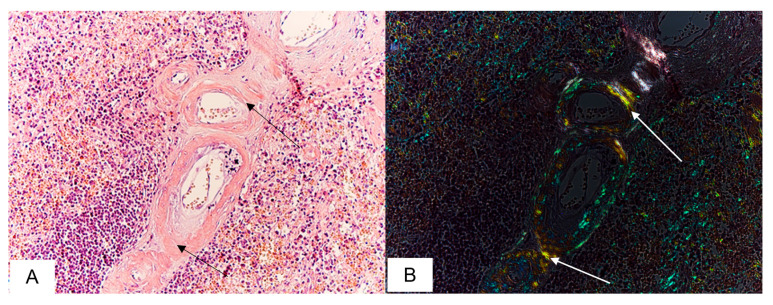
Microscopic sample of spleen tissue of the deceased female patient, 77 years of age. Day 13 from the illness onset. Massive amyloid depositions in the splenic vessels (black arrows) (**A**), displaying apple-green birefringence under polarized light (white arrows) (**B**). Staining with CR, ×200.

On macroscopic assessment, the spleen weight ranged from 90 to 200 g. In most autopsies, the spleen had a firm consistency, a smooth capsule, and a dark cherry or red-brown cut surface. A sebaceous glow on the cut surface was observed in 5 cases. An encapsulated cyst of 1.2 cm in diameter filled with clear yellowish fluid was found in one of the performed autopsies. Also, a single case of focal triangular infarction was reported. Microscopic findings included congestion of the splenic red pulp with fibrosis foci and thickening of the arteriolar walls due to sclerosis and hyalinosis. Amyloid depositions were detected in the red pulp and within the walls of the central arteries supplying the spleen follicles (Figure 5). In some cases, the splenic structural organization was affected by massive abnormal fibrous deposits.

### 3.6. Immunohistochemical Analysis

**Figure 6:**
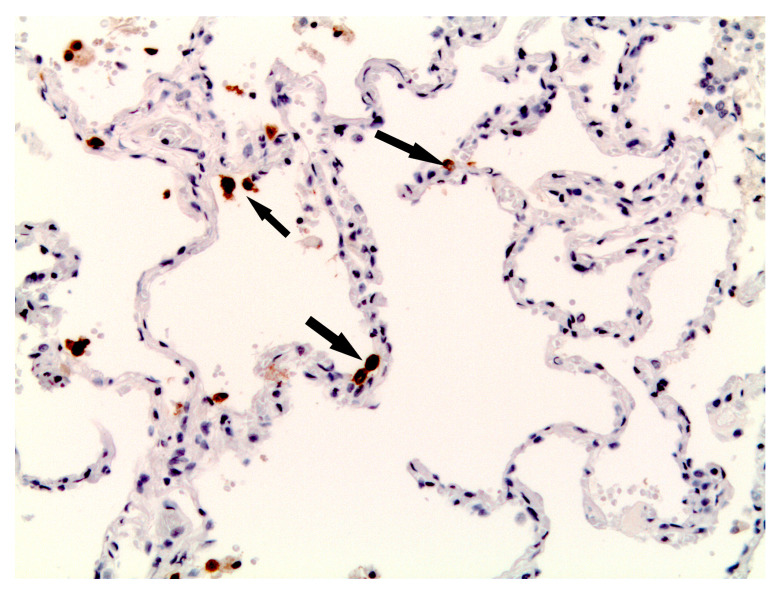
Microscopic sample of lung tissue of the deceased male patient, 72 years of age. Day 10 from the illness onset. IHC antigen-antibody reaction in type II alveolocytes (black arrows), using an antibody to the SARS-CoV-2 S-protein, ×200.

In 13 (59%) observations, immunohistochemical staining with an antibody to SARS-CoV-2 S-protein revealed positive immune reactions in type II alveolocytes (Figure 6). 

Based on immunohistochemical typing of amyloid, the diagnosis of AA amyloidosis was confirmed in 5 (23%) cases, AL lambda–in 7 (32%), AL kappa–in 2 (9%), and ATTR–in 8 (36%) cases (Table 2).

**Figure 7:**
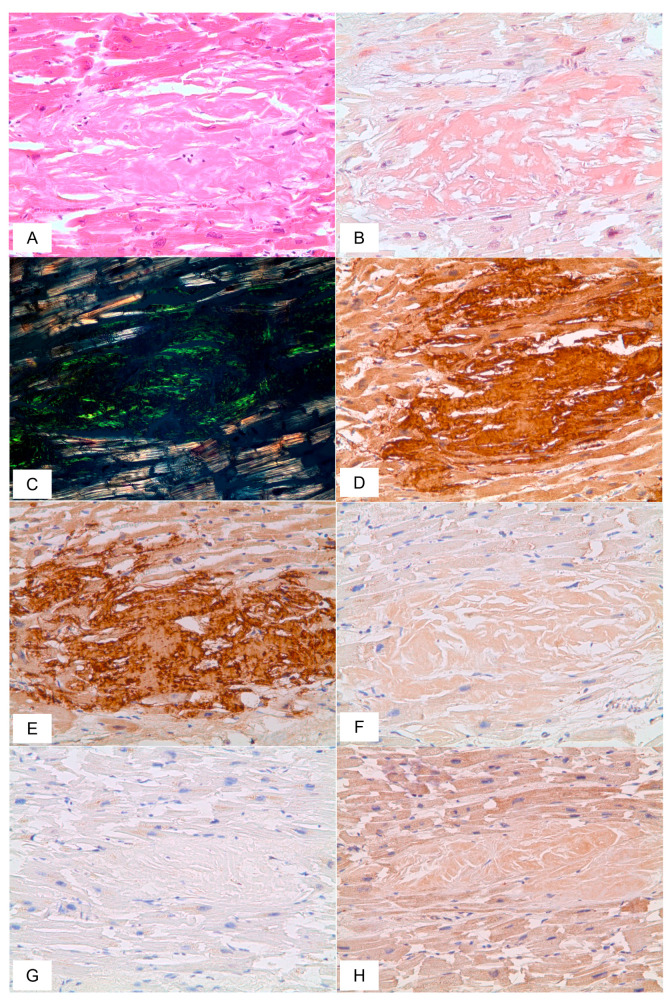
Microscopic sample of heart tissue of the deceased male patient, 89 years of age. Day 9 from the illness onset. Histological staining with H&E showed deposits of homogenous eosinophilic structures (**A**). Polarized light microscopy of the specimen stained with CR demonstrated a green birefringence under polarized light which is specific for amyloid (**B**,**C**). Positive immunostaining with the antibodies to amyloid P-component (**D**), anti-TTR antibody (**E**), and Negative immunostaining was observed for anti-κ-light chain (**F**), AA amyloid (**G**), and anti-λ-light chain (**H**) antibodies. (**A**–**H**) ×200.

The diagnosis of AA amyloidosis was established in patients with such primary medical conditions as periodic disease, arterial hypertension with renal involvement, chronic interstitial nephritis, and atherosclerotic encephalopathy. AL lambda amyloidosis was found in patients with the underlying diagnosis of systemic amyloidosis, multiple myeloma (MM), and novel coronavirus infection. COVID-19 was considered a primary disease in all patients with transthyretin amyloidosis. However, the presence of ATTR in these cases was determined only at autopsy (Figure 7).

## 4. Discussion

Initially, it was believed that novel coronavirus disease 2019 primarily affects the respiratory system, but later COVID-19 was proven to be a complex disorder involving multiple system organs and having a wide range of extrapulmonary manifestations [18,19,20]. Thus, there is a common point between COVID-19 and systemic amyloidosis regarding the multiorgan involvement in the pathological process. The significant overlap in the comorbidities observed in systemic amyloidosis and those associated with COVID-19 leads to a heightened risk for severe morbidity and mortality in amyloidosis patients who contracted COVID-19. Detecting amyloidosis is a challenging process as most symptoms at its early stages are nonspecific, and therefore the diagnosis is often delayed [21,22,23]. If patients with underlying amyloidosis remain undiagnosed and contract COVID-19, the impact of amyloid multiorgan deposition would not be considered in their clinical management. This factor may dramatically increase the risk for fatal outcomes in this group of patients.

Our study focuses on the pathomorphological analysis of autopsy records from deceased persons with underlying systemic amyloidosis which increased COVID-19 disease severity.

As known, COVID-19 is characterized by a broad spectrum of respiratory disorders, ranging from mild upper airway symptoms to progressive viral pneumonia with labored breathing and hypoxemia [24,25]. The key pathologic findings in the lungs comprise different stages of bilateral DAD [26]. In our study, the histopathologic patterns of acute- and chronic-phase DAD were similar to those described in the previous studies elucidating COVID-19 pathology [27,28,29,30,31,32]. The exudative phase of DAD was characterized by hyaline membrane formation, congestion of the alveolar septal capillaries, interstitial edema, interstitial lymphocytic inflammation, and alveolar epithelial desquamation. The organizing phase of DAD included the fibroblastic proliferation within the airspaces and alveolar septa with mild to moderate interstitial lymphocytic infiltrates. The most common histological findings of the proliferative phase of DAD were type II pneumocyte hyperplasia, squamous metaplasia, and multinucleated cells.

The respiratory distress caused by COVID-19 may have a severe impact on patients with systemic amyloidosis, as amyloid fibril infiltration can be found throughout the respiratory tract at autopsy, and patients with cardiac involvement of amyloidosis may have a pre-existing chronic elevation of pulmonary venous pressure. In our study, we identified 8 cases of alveolar-septal amyloidosis which was accompanied by massive intravascular amyloid deposits. Pulmonary impairment due to alveolar-septal amyloidosis is rarely the dominant clinical manifestation and, as a result, it is mostly diagnosed post-mortem [33,34].

The presence of pre-existing cardiovascular disorders seems to be linked with an increased risk of more severe novel coronavirus infection. High mortality rates are reported among patients with chronic heart failure, ischemic heart disease, cardiosclerosis, and arterial hypertension [35,36]. The most common cardiovascular manifestations associated with COVID-19 include myocarditis, acute myocardial infarction, cardiac arrythmia, and acute heart failure [37,38,39]. Macroscopic findings of the heart injury described in the literature comprise ventricular hypertrophy and dilatation [40]. In our study, the signs of right ventricular hypertrophy were observed in 8% of autopsies, while those of left ventricular hypertrophy–in 81% of cases.

Cardiac complications caused by infection with SARS-CoV-2 are deemed to be associated with both direct and indirect viral effects on the myocardium. The direct mechanism of myocardial injury is promoted by a viral invasion of cardiomyocytes via the viral S-protein, a type of transmembrane glycoprotein, and the interaction of the virus with angiotensin-converting enzyme (ACE) 2 present in cardiac cells [41,42,43]. However, the viral entry into cardiomyocytes depends not only on the viral S protein binding to the host-cell receptors. As known, the S protein critically depends on priming by host cell proteases. According to Hoffman et al. [44], the SARS-CoV-2 virus uses ACE2 for entry into cells and the transmembrane protease serine 2 for S protein priming.

The mechanism of indirect myocardial injury is related to cytokine storm due to hyperactivation of the immune system [45]. While most mildly ill COVID-19 patients exhibit a normal immune response and ultimately effectively eliminate the virus, patients with severe disease associated with multiple comorbidities may experience lymphopenia and overactive immune response leading to the hyper-induction and release of cytokines [46,47]. 

Both mechanisms of the myocardial injury caused by the SARS-CoV-2 virus are interrelated, as the direct damage of cardiomyocytes leads to the hyperactivation of the inflammatory response. At the same time, the induction and release of inflammatory cytokines cause apoptosis and necrosis of cardiomyocytes, thus aggravating cardiovascular conditions [48]. 

Patients with systemic AL amyloidosis who receive immunosuppressive drugs can exhibit more severe manifestations of cytokine storm leading to serious complications and even death [49]. Patients with ATTR amyloidosis can also have elevated levels of proinflammatory cytokines at baseline. Since their immune system is often weakened because of age or chronic illness, these patients are at increased risk for adverse outcomes after contracting SARS-CoV-2 infection [50].

Cardiac amyloidosis is a rare but serious disease complicating the clinical pattern of COVID-19. There are published cases of transthyretin amyloidosis diagnosed only at autopsy in elderly patients who died from the novel coronavirus disease. As shown by Menter et al., transthyretin amyloidosis was found at post-mortem examination in 6 of 21 (28%) persons deceased from COVID-19 [51]. They had a severe form of cardiomegaly, and histopathological analysis revealed diffusive and focal amyloid deposits in the interstitial tissue and vascular walls. In our study, amyloid depositions in the heart were found in 86% of patients. Autopsy findings comprised cardiomyocyte atrophy due to the formation of amyloid foci in the myocardium and massive intravascular amyloid accumulations. These lesions, in combination with direct injury to the cardiac muscle by SARS-CoV-2, caused cardiovascular complications leading to the patient’s death. 

The interplay between amyloidosis and COVID-19 may be associated with a higher risk of renal failure that could impact the illness severity [34]. Amyloid deposits in the kidney can cause various renal manifestations, including albuminuria, azotemia, and renal dysfunction [52,53]. If systemic amyloidosis is coupled with cardiovascular comorbidities such as heart failure, the risk of renal dysfunction increases. In our study, massive renal amyloid deposits were observed in 73% of patients who contracted COVID-19, and, as a result, experienced more severe clinical manifestations of the disease. Whether SARS-CoV-2 infection directly impacts the homeostasis of the kidney or is a result of multi-organ failure remains poorly understood; however acute kidney injury is commonly reported in patients with severe COVID-19 [54,55,56].

Only a few scientific publications are available describing specific clinical characteristics of COVID-19 in large cohorts of patients with pre-existing systemic amyloidosis. Lewis et al. [57], evaluated a cohort of 152 ATTR patients and 103 AL amyloidosis patients during the period between January 2020 and April 2022. They concluded that patients with amyloidosis had an increased risk of severe infection and fatal outcomes from COVID-19, especially those of older age and receiving active immunotherapy. They also emphasized that there was an excess mortality of 128% in the ATTR cohort and 75% in the AL amyloidosis cohort in 2021 when comparing the mortality of amyloidosis with pre-pandemic years. 

As highlighted in the literature, the strongest risk factors for COVID-19 mortality were pre-existing comorbidities like MM and systemic amyloidosis. Wood et al. [58] evaluated the outcomes of 250 patients with hematologic disorders, including a subgroup of 40 patients with multiple MM or systemic AL amyloidosis. In this subgroup, 81% of patients had moderate or severe COVID-19 infection. All patients needed hospital admission and those with severe infection required intensive care unit admission. The mortality rate in the hospitalized patients reached 28%. 

A much larger cohort of 9225 patients with MM or AL amyloidosis was evaluated by Ho et al. [59] in a multi-center study. Within the AL amyloidosis subgroup, 13 patients were affected by COVID-19, and hospital admission was required only for 4 (30.8%) of these patients. Severe manifestations of the disease developed in 3 of them (23.1%), and one patient (7.8%) needed intensive care unit (ICU) admission. 

Our study included only one patient with MM who had cardio-, spleno- and hepatomegaly. The patient died from multiple myeloma coupled with COVID-19, primarily affecting the pulmonary system. The illness was complicated by systemic AL amyloidosis, bilateral pneumonia, and multiorgan failure which was considered the major cause of death.

## 5. Conclusions

Our analysis has elucidated a greater severity of COVID-19 in patients with pre-existing systemic amyloidosis which aggravated the viral impact on the host’s body, enhancing both pulmonary and extrapulmonary presentations of the disease that led to multiorgan failure and patient’s death.

The multiorgan involvement detected within our study in patients with systemic amyloidosis who contract COVID-19 is linked to a higher risk for more severe illness. When such patients with undiagnosed amyloidosis are admitted to the ICU, the treatment is administered without considering the pre-existing condition. As a result, these patients are at increased risk of getting severe complications or dying.

In patients with the established diagnosis of AL amyloidosis, the risk of severe COVID-19 is associated with the administration of regular courses of immunosuppressive anti-plasma cell therapy which can cause the hyperactivation of the immune system and the clinical manifestations of cytokine storm, which, in turn, appears to be critical in the development of the worst consequences. 

## Figures and Tables

**Table 1:** Microscopic changes in the lungs of patients with COVID-19 and pre-existing systemic amyloidosis.

Finding	Number of Cases	%
Pulmonary capillary congestion	22/22	100
Hyaline membranes	17/22	77
Fibrin in alveoli	18/22	81
Interstitial edema	9/22	41
Metaplasia of alveolar epithelium	6/22	27
Desquamation of alveolar epithelium	19/22	86
Pulmonary infarction	2/22	9
Microvascular thrombosis	16/22	72
Giant cells	8/22	36
Hyperplasia of Type II alveolocytes	10/22	45
Pleuritis with fibrin	2/22	9
Amyloid deposits	8/22	36

**Table 2:** Correlation of patient age and gender with amyloid type and localization.

Patient No.	Gender	Age	Amyloid Type	Heart	Lung	Kidney	Liver	Spleen	Pancreas	Gastrointestinal Tract	Thyroid Gland	Adrenal Gland
1	m	56	AA	*		*	*	*				
2	f	63	AA			*		*		*		
3	m	53	AA	*	*	*	*	*		*	*	*
4	m	71	AA	*		*	*	*	*	*		
5	f	80	AA			*			*	*	*	*
6	m	84	AL-lambda	*		*	*	*				
7	f	73	AL-lambda	*		*		*				
8	m	70	AL-lambda	*			*	*		*		*
9	f	69	AL-lambda	*		*	*		*	*		
10	f	87	AL-lambda	*			*			*		*
11	m	59	AL-lambda	*	*	*				*		
12	f	60	AL-lambda			*		*		*		
13	f	75	AL-kappa	*	*	*	*	*	*	*	*	
14	m	88	AL-kappa	*	*			*		*		
15	f	89	ATTR	*		*				*		
16	f	80	ATTR	*	*					*		
17	m	88	ATTR	*		*	*	*				
18	f	92	ATTR	*		*				*		
19	m	95	ATTR	*	*			*				
20	m	91	ATTR	*	*		*			*		
21	m	95	ATTR	*		*		*		*		
22	f	93	ATTR	*	*	*			*			
Organ involvement in amyloidosis (%) revealed by 22 autopsies				86	36	73	45	59	23	73	14	18

* The organs where amyloid deposits were found.

## Data Availability

All data and materials are available upon reasonable request. Address to Z.G. (email: gioeva_z@mail.ru) or L.M. (email: mikhalevalm@yandex.ru), Avtsyn Research Institute of Human Morphology of Federal State Budgetary Scientific Institution “Petrovsky National Research Centre of Surgery”, Moscow, Russian Federation.

## Abbreviations

AA, amyloid A protein; ACE, angiotensin-converting enzyme; AL, amyloid light chain; ATTR, amyloid transthyretin; COVID-19, Coronavirus-19 disease; CR, Congo red; DAD, diffuse alveolar damage; H&E, hematoxylin plus eosin; ICU, intensive care unit; IHC, immunohistochemistry; MM, multiple myeloma; PCR, polymerase-chain-reaction; RNA, ribonucleic acid; S protein, viral spike protein; SARS-CoV-2, Severe acute respiratory syndrome coronavirus 2; WHO, World Health Organization.
